# Supplementary material for: HIVprotI: an integrated web based platform for prediction and design of HIV proteins inhibitors
Source: J Cheminform. 2018 Mar 9;10:12. doi: 10.1186/s13321-018-0266-y (PMC5845081; doi:10.1186/s13321-018-0266-y)
Supplement: Supplementary file 2 — Additional file 2. Source code of HIVProtI web server. [file 13321_2018_266_MOESM2_ESM.zip › HIVprotI_Source-code/hivprotI/weka-3-6-6/doc/weka/gui/explorer/ExplorerDefaults.html]

ExplorerDefaults


---


|  |  |  |
| --- | --- | --- |
| **Overview** \| **Package** \| **Class** \| **Tree** \| **Deprecated** \| **Index** \| **Help** \| **Weka's home** | |  |
| **PREV CLASS**   **NEXT CLASS** | **FRAMES**    **NO FRAMES**     **All Classes** |  |
| SUMMARY: NESTED \| FIELD \| CONSTR \| METHOD | DETAIL: FIELD \| CONSTR \| METHOD |  |


---


## weka.gui.explorer Class ExplorerDefaults

```
java.lang.Object
  weka.gui.explorer.ExplorerDefaults
```

**All Implemented Interfaces:**: java.io.Serializable

---

``` public class ExplorerDefaults extends java.lang.Object implements java.io.Serializable ```

This class offers get methods for the default Explorer settings in
the props file `weka/gui/explorer/Explorer.props`.

**Version:**
:   $Revision: 7059 $

**Author:**
:   FracPete (fracpete at waikato dot ac dot nz)

**See Also:**: `PROPERTY_FILE`, Serialized Form

---

| **Field Summary** | |
| --- | --- |
| `static java.lang.String` | `PROPERTY_FILE`             The name of the properties file. |


**Constructor Summary**

`ExplorerDefaults()`


| **Method Summary** | |
| --- | --- |
| `static java.lang.String` | `get(java.lang.String property, java.lang.String defaultValue)`             returns the value for the specified property, if non-existent then the default value. |
| `static int` | `getASCrossvalidationFolds()`             returns the default number of folds of the CV in the attribute selection panel. |
| `static java.lang.Object` | `getASEvaluator()`             returns the default attribute evalautor (fully configured) for the attribute selection panel. |
| `static int` | `getASRandomSeed()`             returns the default random seed value in the attribute selection panel. |
| `static java.lang.Object` | `getASSearch()`             returns the default attribute selection search scheme (fully configured) for the attribute selection panel. |
| `static java.lang.Object` | `getAssociator()`             returns the default associator (fully configured) for the associations panel. |
| `static int` | `getASTestMode()`             returns the default attribute selection test mode for the attribute selection panel. |
| `static java.lang.Object` | `getClassifier()`             returns the default classifier (fully configured) for the classify panel. |
| `static boolean` | `getClassifierCostSensitiveEval()`             returns whether the evaluation of the classifier is done cost-sensitively. |
| `static int` | `getClassifierCrossvalidationFolds()`             returns the default number of folds of the CV in the classify panel. |
| `static java.lang.String` | `getClassifierOutputAdditionalAttributes()`             returns the string with the additional indices to output alongside the predictions. |
| `static boolean` | `getClassifierOutputConfusionMatrix()`             returns whether the confusion matrix for the classifier is output. |
| `static boolean` | `getClassifierOutputEntropyEvalMeasures()`             returns whether entropy-based evaluation meastures of the classifier are output. |
| `static boolean` | `getClassifierOutputModel()`             returns whether the built model is output. |
| `static boolean` | `getClassifierOutputPerClassStats()`             returns whether additional per-class stats of the classifier are output. |
| `static boolean` | `getClassifierOutputPredictions()`             returns whether the predictions of the classifier are output as well. |
| `static boolean` | `getClassifierOutputSourceCode()`             returns whether the source of a sourcable Classifier is output in the classify tab. |
| `static int` | `getClassifierPercentageSplit()`             returns the default classifier test mode for the classify panel (0-99). |
| `static boolean` | `getClassifierPreserveOrder()`             returns whether the order is preserved in case of the percentage split in the classify tab. |
| `static int` | `getClassifierRandomSeed()`             returns the default random seed value for the classifier for the classify panel. |
| `static java.lang.String` | `getClassifierSourceCodeClass()`             returns the default classname for a sourcable Classifier in the classify tab. |
| `static boolean` | `getClassifierStorePredictionsForVis()`             returns whether the predictions of the classifier are stored for visualization. |
| `static int` | `getClassifierTestMode()`             returns the default classifier test mode for the classify panel. |
| `static java.lang.Object` | `getClusterer()`             returns the default clusterer (fully configured) for the clusterer panel. |
| `static boolean` | `getClustererStoreClustersForVis()`             returns whether the clusters are storeed for visualization purposes in the cluster panel. |
| `static int` | `getClustererTestMode()`             returns the default cluster test mode for the cluster panel. |
| `static java.lang.Object` | `getFilter()`             returns the default filter (fully configured) for the preprocess panel. |
| `static boolean` | `getInitGenericObjectEditorFilter()`             returns if the GOEs in the Explorer will be initialized based on the data that is loaded into the Explorer. |
| `static java.lang.String` | `getInitialDirectory()`             Returns the initial directory for the file chooser used for opening datasets. |
| `static java.util.Properties` | `getProperties()`             returns the associated properties file. |
| `static java.lang.String[]` | `getTabs()`             returns an array with the classnames of all the additional panels to display as tabs in the Explorer. |
| `static void` | `main(java.lang.String[] args)`             only for testing - prints the content of the props file. |

**Methods inherited from class java.lang.Object**

`equals, getClass, hashCode, notify, notifyAll, toString, wait, wait, wait`

**Field Detail**

### PROPERTY\_FILE

```
public static final java.lang.String PROPERTY_FILE
```

:   The name of the properties file.

    **See Also:**: Constant Field Values


**Constructor Detail**

### ExplorerDefaults

```
public ExplorerDefaults()
```


**Method Detail**

### get

```
public static java.lang.String get(java.lang.String property,
                                   java.lang.String defaultValue)
```

:   returns the value for the specified property, if non-existent then the
    default value.

    :   **Parameters:**: `property` - the property to retrieve the value for: `defaultValue` - the default value for the property **Returns:**: the value of the specified property

---


### getProperties

```
public static final java.util.Properties getProperties()
```

:   returns the associated properties file.

    :   **Returns:**: the props file

---


### getInitGenericObjectEditorFilter

```
public static boolean getInitGenericObjectEditorFilter()
```

:   returns if the GOEs in the Explorer will be initialized based on the
    data that is loaded into the Explorer.

    :   **Returns:**: true if the GOEs get initialized

---


### getTabs

```
public static java.lang.String[] getTabs()
```

:   returns an array with the classnames of all the additional panels to
    display as tabs in the Explorer.

    :   **Returns:**: the classnames

---


### getInitialDirectory

```
public static java.lang.String getInitialDirectory()
```

:   Returns the initial directory for the file chooser used for opening
    datasets.

    The following placeholders are recognized:

    ```
       %t - the temp directory
       %h - the user's home directory
       %c - the current directory
       %% - gets replaced by a single percentage sign
    ```

    :   **Returns:**: the default directory

---


### getFilter

```
public static java.lang.Object getFilter()
```

:   returns the default filter (fully configured) for the preprocess panel.

    :   **Returns:**: the default filter, null if none

---


### getClassifier

```
public static java.lang.Object getClassifier()
```

:   returns the default classifier (fully configured) for the classify panel.

    :   **Returns:**: the default classifier, ZeroR by default

---


### getClassifierTestMode

```
public static int getClassifierTestMode()
```

:   returns the default classifier test mode for the classify panel.

    :   **Returns:**: the default test mode

---


### getClassifierCrossvalidationFolds

```
public static int getClassifierCrossvalidationFolds()
```

:   returns the default number of folds of the CV in the classify panel.

    :   **Returns:**: the default number of folds

---


### getClassifierPercentageSplit

```
public static int getClassifierPercentageSplit()
```

:   returns the default classifier test mode for the classify panel (0-99).

    :   **Returns:**: the default precentage split

---


### getClassifierOutputModel

```
public static boolean getClassifierOutputModel()
```

:   returns whether the built model is output.

    :   **Returns:**: true if the built model is output

---


### getClassifierOutputPerClassStats

```
public static boolean getClassifierOutputPerClassStats()
```

:   returns whether additional per-class stats of the classifier are output.

    :   **Returns:**: true if stats are output

---


### getClassifierOutputEntropyEvalMeasures

```
public static boolean getClassifierOutputEntropyEvalMeasures()
```

:   returns whether entropy-based evaluation meastures of the classifier
    are output.

    :   **Returns:**: true if output

---


### getClassifierOutputConfusionMatrix

```
public static boolean getClassifierOutputConfusionMatrix()
```

:   returns whether the confusion matrix for the classifier is output.

    :   **Returns:**: true if matrix is output

---


### getClassifierOutputPredictions

```
public static boolean getClassifierOutputPredictions()
```

:   returns whether the predictions of the classifier are output as well.

    :   **Returns:**: true if predictions are output as well

---


### getClassifierOutputAdditionalAttributes

```
public static java.lang.String getClassifierOutputAdditionalAttributes()
```

:   returns the string with the additional indices to output alongside the
    predictions.

    :   **Returns:**: the indices, 0 if none are output

---


### getClassifierStorePredictionsForVis

```
public static boolean getClassifierStorePredictionsForVis()
```

:   returns whether the predictions of the classifier are stored for
    visualization.

    :   **Returns:**: true if predictions are stored

---


### getClassifierCostSensitiveEval

```
public static boolean getClassifierCostSensitiveEval()
```

:   returns whether the evaluation of the classifier is done cost-sensitively.

    :   **Returns:**: true if cost-sensitively done

---


### getClassifierRandomSeed

```
public static int getClassifierRandomSeed()
```

:   returns the default random seed value for the classifier for the
    classify panel.

    :   **Returns:**: the default random seed

---


### getClassifierPreserveOrder

```
public static boolean getClassifierPreserveOrder()
```

:   returns whether the order is preserved in case of the percentage split
    in the classify tab.

    :   **Returns:**: true if order is preserved

---


### getClassifierOutputSourceCode

```
public static boolean getClassifierOutputSourceCode()
```

:   returns whether the source of a sourcable Classifier is output
    in the classify tab.

    :   **Returns:**: true if the source code is output

---


### getClassifierSourceCodeClass

```
public static java.lang.String getClassifierSourceCodeClass()
```

:   returns the default classname for a sourcable Classifier in the classify tab.

    :   **Returns:**: the default classname

---


### getClusterer

```
public static java.lang.Object getClusterer()
```

:   returns the default clusterer (fully configured) for the clusterer panel.

    :   **Returns:**: the default clusterer, EM by default

---


### getClustererTestMode

```
public static int getClustererTestMode()
```

:   returns the default cluster test mode for the cluster panel.

    :   **Returns:**: the default test mode

---


### getClustererStoreClustersForVis

```
public static boolean getClustererStoreClustersForVis()
```

:   returns whether the clusters are storeed for visualization purposes
    in the cluster panel.

    :   **Returns:**: true if clusters are stored

---


### getAssociator

```
public static java.lang.Object getAssociator()
```

:   returns the default associator (fully configured) for the associations panel.

    :   **Returns:**: the default associator, Apriori by default

---


### getASEvaluator

```
public static java.lang.Object getASEvaluator()
```

:   returns the default attribute evalautor (fully configured) for the
    attribute selection panel.

    :   **Returns:**: the default attribute evaluator, CfsSubsetEval by default

---


### getASSearch

```
public static java.lang.Object getASSearch()
```

:   returns the default attribute selection search scheme (fully configured)
    for the attribute selection panel.

    :   **Returns:**: the default search scheme, BestFirst by default

---


### getASTestMode

```
public static int getASTestMode()
```

:   returns the default attribute selection test mode for the attribute
    selection panel.

    :   **Returns:**: the default test mode

---


### getASCrossvalidationFolds

```
public static int getASCrossvalidationFolds()
```

:   returns the default number of folds of the CV in the attribute selection
    panel.

    :   **Returns:**: the default number of folds

---


### getASRandomSeed

```
public static int getASRandomSeed()
```

:   returns the default random seed value in the attribute selection panel.

    :   **Returns:**: the default random seed

---


### main

```
public static void main(java.lang.String[] args)
```

:   only for testing - prints the content of the props file.

    :   **Parameters:**: `args` - commandline parameters - ignored


---


|  |  |  |
| --- | --- | --- |
| **Overview** \| **Package** \| **Class** \| **Tree** \| **Deprecated** \| **Index** \| **Help** \| **Weka's home** | |  |
| **PREV CLASS**   **NEXT CLASS** | **FRAMES**    **NO FRAMES**     **All Classes** |  |
| SUMMARY: NESTED \| FIELD \| CONSTR \| METHOD | DETAIL: FIELD \| CONSTR \| METHOD |  |


---
